# C-Reactive Protein-to-Albumin Ratio in Pediatric Inflammatory Bowel Disease: Associations with Disease Activity and Comparison with C-Reactive Protein Alone

**DOI:** 10.3390/diagnostics16162608

**Published:** 2026-08-17

**Authors:** Jihye Kim, You Ie Kim, Sang Yong Kim

**Affiliations:** Department of Pediatrics, Incheon St. Mary’s Hospital, College of Medicine, The Catholic University of Korea, 56 Dongsu-ro, Bupyeong-gu, Incheon 21431, Republic of Korea; etoile0510@gmail.com (J.K.); pedksy@naver.com (S.Y.K.)

**Keywords:** Crohn’s disease, ulcerative colitis, pediatrics, C-reactive protein-to-albumin ratio, biomarker, disease activity, inflammatory bowel disease

## Abstract

**Background/Objectives:** The C-reactive protein-to-albumin ratio (CAR) is an emerging biomarker of disease activity in adult inflammatory bowel disease; pediatric data remain limited. We evaluated CAR in pediatric Crohn’s disease (CD) versus ulcerative colitis (UC). **Methods:** We retrospectively reviewed 97 consecutive pediatric patients (79 CD, 18 UC) diagnosed at a single center between 2012 and 2023. **Results:** CAR was higher in CD (median 10.06) than UC (0.49; *p* < 0.001). In CD, CAR correlated with erythrocyte sedimentation rate, fecal calprotectin, and clinical and endoscopic disease activity (all *p* < 0.05), differed across severity categories (*p* = 0.001 clinical, *p* = 0.003 endoscopic), and was higher without perianal disease (*p* = 0.005); the corresponding analyses in UC (*n* = 18) were underpowered and did not reach significance. Receiver operating characteristic analysis yielded an area under the curve (AUC) of 0.840 (cut-off 1.62; sensitivity 86.1%, specificity 72.2%), not exceeding that of CRP alone (AUC 0.851; DeLong *p* = 0.090). CAR decreased significantly from diagnosis to one-year follow-up in both remission and relapse groups (both *p* < 0.001). **Conclusions:** CAR reflects disease severity in pediatric CD and responds to treatment, but did not outperform CRP alone for distinguishing CD from UC. Its plausible value lies in activity assessment and serial monitoring rather than in diagnostic discrimination, a hypothesis that the present design cannot test.

## 1. Introduction

Inflammatory bowel disease (IBD), which encompasses Crohn’s disease (CD) and ulcerative colitis (UC), is a chronic inflammatory disorder of the gastrointestinal tract characterized by a relapsing and remitting clinical course. The incidence of pediatric IBD has been steadily increasing worldwide, with pediatric-onset disease often exhibiting a more extensive distribution and more aggressive course than adult-onset disease. Chronic intestinal inflammation in pediatric patients may also impair growth and nutritional status, posing additional clinical challenges in this population [1,2]. A systematic review of population-based studies spanning the first two decades of the twenty-first century has confirmed rising incidence and prevalence of pediatric-onset IBD across most world regions [3], and nationwide Korean data have documented a parallel increase in pediatric IBD prevalence together with a marked expansion in anti-tumor necrosis factor use over the same period [4].

The definitive diagnosis of IBD requires invasive procedures, including esophagogastroduodenoscopy and ileocolonoscopy with biopsies, which are particularly challenging in pediatric patients owing to the need for sedation and procedural burden. Periodic endoscopic surveillance is often required during follow-up to assess disease activity and treatment response. Consequently, there is a growing clinical need for reliable, non-invasive, and cost-effective biomarkers to support clinical decision-making in pediatric IBD, both at diagnosis and during long-term follow-up [5,6]. This need is reinforced by current treat-to-target frameworks: the ECCO-ESPGHAN guideline for paediatric Crohn’s disease recommends serial biomarker monitoring to guide treatment decisions [7], and the STRIDE-II consensus explicitly designates normalization of CRP and fecal calprotectin as an intermediate treatment target in both adult and pediatric IBD [8].

C-reactive protein (CRP) is an acute-phase protein produced by the liver in response to systemic inflammation, while serum albumin is a negative acute-phase protein whose synthesis is downregulated during inflammation and also reflects nutritional status. Both CRP and albumin are routinely measured, inexpensive, widely available, and rapidly assessable. The CRP-to-albumin ratio (CAR), which integrates these complementary biomarkers, has been reported as a prognostic marker in various inflammatory and non-inflammatory diseases, including cardiovascular, respiratory, infectious, rheumatologic, oncologic, and renal disorders [9,10].

In adult patients with IBD, several studies have demonstrated the clinical utility of CAR in monitoring disease activity, predicting treatment response, and assessing prognosis. Qin et al. first reported CAR as a useful biomarker in adult CD [11], and subsequent studies have further validated its role in both CD and UC [12,13]. Data on CAR in pediatric IBD remain limited. A multi-center study by Glapa-Nowak et al. reported that CAR at diagnosis could differentiate severe from quiescent CD in pediatric patients, with moderate ability to differentiate penetrating from non-penetrating disease behavior [14]. Several of these adult studies compared CAR with CRP measured alone, but that comparison has not been made in children, and no previous study in either population has tested whether CAR discriminates CD from UC better than CRP alone.

We therefore aimed to evaluate the utility of CAR as a non-invasive biomarker in pediatric IBD. The primary objective was to assess the diagnostic performance of CAR in differentiating CD from UC. Secondary objectives were to investigate the association of CAR with clinical and endoscopic disease activity, disease behavior, and clinical outcomes during one-year follow-up.

## 2. Materials and Methods

### 2.1. Study Design and Patient Selection

**Figure 1:**
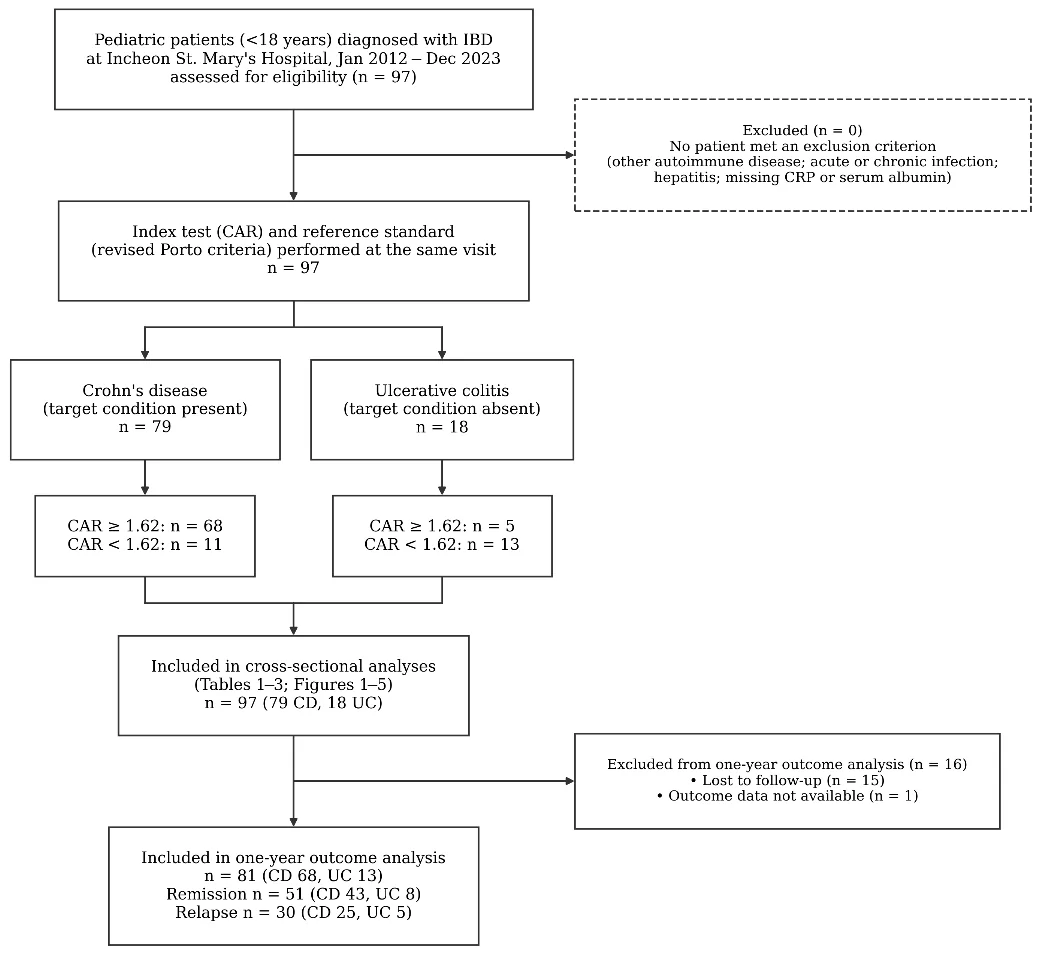
Flow of participants. The index test (CAR) and the reference standard (revised Porto criteria) were obtained at the same visit, with no intervening treatment. CAR, C-reactive protein-to-albumin ratio; CD, Crohn’s disease; CRP, C-reactive protein; IBD, inflammatory bowel disease; UC, ulcerative colitis.

We retrospectively reviewed the medical records of pediatric patients younger than 18 years diagnosed with IBD at Incheon St. Mary’s Hospital between January 2012 and December 2023. IBD was diagnosed and classified into CD and UC based on the revised Porto criteria [5], through comprehensive evaluation of clinical features, laboratory findings, endoscopic findings with histopathology, and radiologic investigations including magnetic resonance enterography. All consecutive eligible patients diagnosed during the study period were included. Patients with other autoimmune diseases, acute or chronic infections, hepatitis, or missing CRP or serum albumin measurements at diagnosis were to be excluded; in the event, no patient met any exclusion criterion, and all 97 consecutive patients diagnosed during the study period were analyzed. No a priori sample size calculation was performed; the sample size was determined by the size of the available cohort. The flow of participants is shown in Figure 1.

Enrolled patients were classified into two subgroups based on diagnosis (CD and UC). CD patients were further classified by disease behavior according to the Paris classification as luminal (B1, inflammatory) or complicated (B2, stricturing; B3, penetrating), with perianal disease noted as a modifier [15]. The study was reported in accordance with the Strengthening the Reporting of Observational Studies in Epidemiology (STROBE) Statement for observational research and, for the diagnostic accuracy analysis (Section 3.5), with the Standards for Reporting of Diagnostic Accuracy Studies (STARD) 2015 guideline; the completed STARD checklist is provided as Supplementary Material.

### 2.2. Data Collection

Demographic and clinical data, including age at diagnosis, sex, anthropometric measurements (height, weight, body mass index [BMI]) with z-scores based on the 2017 Korean National Growth Charts [16], family history, and surgical history, were collected. Clinical symptoms and the presence of perianal disease, luminal stricture, and luminal fistula were also reviewed. Disease location was classified according to the Paris classification: L1 (distal ileum ± limited cecal disease), L2 (colonic), L3 (ileocolonic), and L4 (upper, proximal to ligament of Treitz) for CD; and E1 (proctitis), E2 (left-sided), E3 (extensive), and E4 (pancolitis) for UC.

### 2.3. Laboratory Findings

Laboratory parameters were collected at IBD diagnosis. Complete blood count (white blood cell [WBC] count, hemoglobin, platelet count), erythrocyte sedimentation rate (ESR), CRP, and serum albumin were analyzed. Fecal calprotectin was measured using a fluorescent enzyme immunoassay (Calprotectin, Phadia AB, Uppsala, Sweden), with the normal range set at ≤50 mg/kg. CAR was calculated by dividing serum CRP (mg/L) by serum albumin (g/dL) at diagnosis.

CRP and serum albumin were measured as part of routine clinical care at the same visit at which the diagnostic endoscopy was performed, so there was no interval between the index test and the reference standard. No patient received corticosteroids, exclusive enteral nutrition, or any other IBD-directed therapy before the index test and the reference standard were obtained; all treatments recorded in this study were started after the diagnosis was established. CAR itself was computed retrospectively for this study, so the treating clinicians were unaware of the CAR value at the time of diagnosis. Conversely, the clinicians who applied the reference standard had access to all routine laboratory results, including CRP and serum albumin, as is unavoidable in routine clinical diagnosis.

### 2.4. Assessment of Clinical and Endoscopic Disease Activity

Clinical disease activity at diagnosis was assessed using the Pediatric Crohn’s Disease Activity Index (PCDAI) [17] for CD and the Pediatric Ulcerative Colitis Activity Index (PUCAI) [18] for UC. CD was classified as mild (<30), moderate (30–37.5), or severe (≥40) by PCDAI; UC as mild (10–34), moderate (35–64), or severe (≥65) by PUCAI.

Endoscopies were performed by pediatric gastroenterologists under conscious sedation with continuous monitoring of vital signs and oxygen saturation. Endoscopic activity was assessed by the Simple Endoscopic Score for Crohn’s Disease (SES-CD) [19] in CD and the Mayo endoscopic subscore [20] in UC. SES-CD was classified as mild (3–6), moderate (7–15), or severe (≥16); the Mayo endoscopic subscore as mild (1), moderate (2), or severe (3). Both CDEIS and SES-CD were recorded for CD; SES-CD was pre-specified as the primary endoscopic index because it is the more widely applied instrument in pediatric CD. One endoscopic subscore entered in the case report form as 4, a value outside the 0–3 range of the instrument, was verified against the endoscopy report and corrected to 3 before analysis.

### 2.5. Treatment and Follow-Up

Treatment modalities, including exclusive enteral nutrition (EEN), 5-aminosalicylic acid (5-ASA), corticosteroids, immunomodulators, and biologic agents, were reviewed. All enrolled patients were scheduled for clinical follow-up for at least one year after the diagnosis of IBD. Complete one-year data were available for 81 of the 97 patients (83.5%), who constituted the population for the one-year outcome analysis presented in Section 3.6; 15 patients were lost to follow-up and 1 had incomplete records. Relapse was defined as clinical exacerbation requiring treatment escalation, including a change to a new treatment regimen or surgery during the one-year follow-up. Treatment changes made for adverse drug effects rather than for disease activity were not counted as relapse.

### 2.6. Statistical Analyses

Statistical analyses were performed using SPSS Statistics version 22.0 (IBM Corp., Armonk, NY, USA), except for the comparison of receiver operating characteristic curves, which was performed in R version 4.3.3 (R Foundation for Statistical Computing, Vienna, Austria) using the pROC package (version 1.18.5). All areas under the curve, their confidence intervals, and the reported accuracy measures were computed in R so that the diagnostic accuracy analysis derives from a single method throughout. The normality of continuous variables was assessed using the Shapiro–Wilk test. Continuous variables are presented as median (range) and were compared using the Mann–Whitney *U* test for two groups and the Kruskal–Wallis test for three or more groups, with Bonferroni-corrected post hoc analyses where appropriate. Categorical variables, presented as numbers (%), were compared using the chi-squared test or Fisher’s exact test as appropriate. Correlations between CAR and other parameters were assessed using Spearman’s rank correlation. Paired comparisons between values at diagnosis and at one-year follow-up were performed using the Wilcoxon signed-rank test. Receiver operating characteristic (ROC) curve analysis evaluated the diagnostic performance of CAR for distinguishing CD from UC, and the optimal cut-off was determined by the maximum Youden index. Because CAR is a composite of CRP and albumin, the area under the curve (AUC) of CAR was compared with that of CRP alone using the DeLong test, in order to establish whether the ratio adds discriminative information beyond its numerator. Sensitivity, specificity, predictive values, and accuracy at the selected cut-off are reported with exact (Clopper–Pearson) binomial 95% confidence intervals (CIs); AUCs are reported with DeLong 95% CIs. Analyses were complete-case; the number of patients contributing to each analysis is given in the tables, and no imputation was performed. Statistical significance was set at *p* < 0.05.

### 2.7. Use of Generative Artificial Intelligence

Generative artificial intelligence (Claude, Anthropic, San Francisco, CA, USA; accessed via claude.ai) was used as an analysis and drafting aid in the preparation of this manuscript. It was used to write and execute the code by which the analyses reported in Section 3 were re-derived from the source dataset (Python 3 with pandas, SciPy and scikit-learn; R 4.3.3 with pROC 1.18.5), to produce Figure 1 by plotting the study’s measured values, to assist in revising the Abstract, Section 3.5, and to verify reference metadata against PubMed records. No artificial intelligence tool was used to generate, fabricate or alter primary data, and no image-generation model was used at any stage; all primary data derive from the institutional case report form, and all figures plot measured values only. The authors made all clinical and scientific judgements, including the case-level classification of relapse and the verification of an out-of-range endoscopic subscore against the source endoscopy report (Section 2.4), verified every reported value against the source data, and take full responsibility for the content and integrity of this manuscript.

## 3. Results

### 3.1. Patient Characteristics

A total of 97 pediatric patients diagnosed with IBD were enrolled, comprising 79 (81.4%) with CD and 18 (18.6%) with UC. Demographic and clinical characteristics are summarized in Table 1.

Male predominance was significantly higher in CD (57/79, 72.2%) than in UC (5/18, 27.8%) (*p* = 0.001). The median age at diagnosis was 14.8 years (range 9.1–18.5) in CD and 12.9 years (range 8.2–17.7) in UC, with no statistically significant difference (*p* = 0.052). Anthropometric measurements, including z-scores for height, weight, and BMI, did not differ significantly between groups.

**Figure 2:**
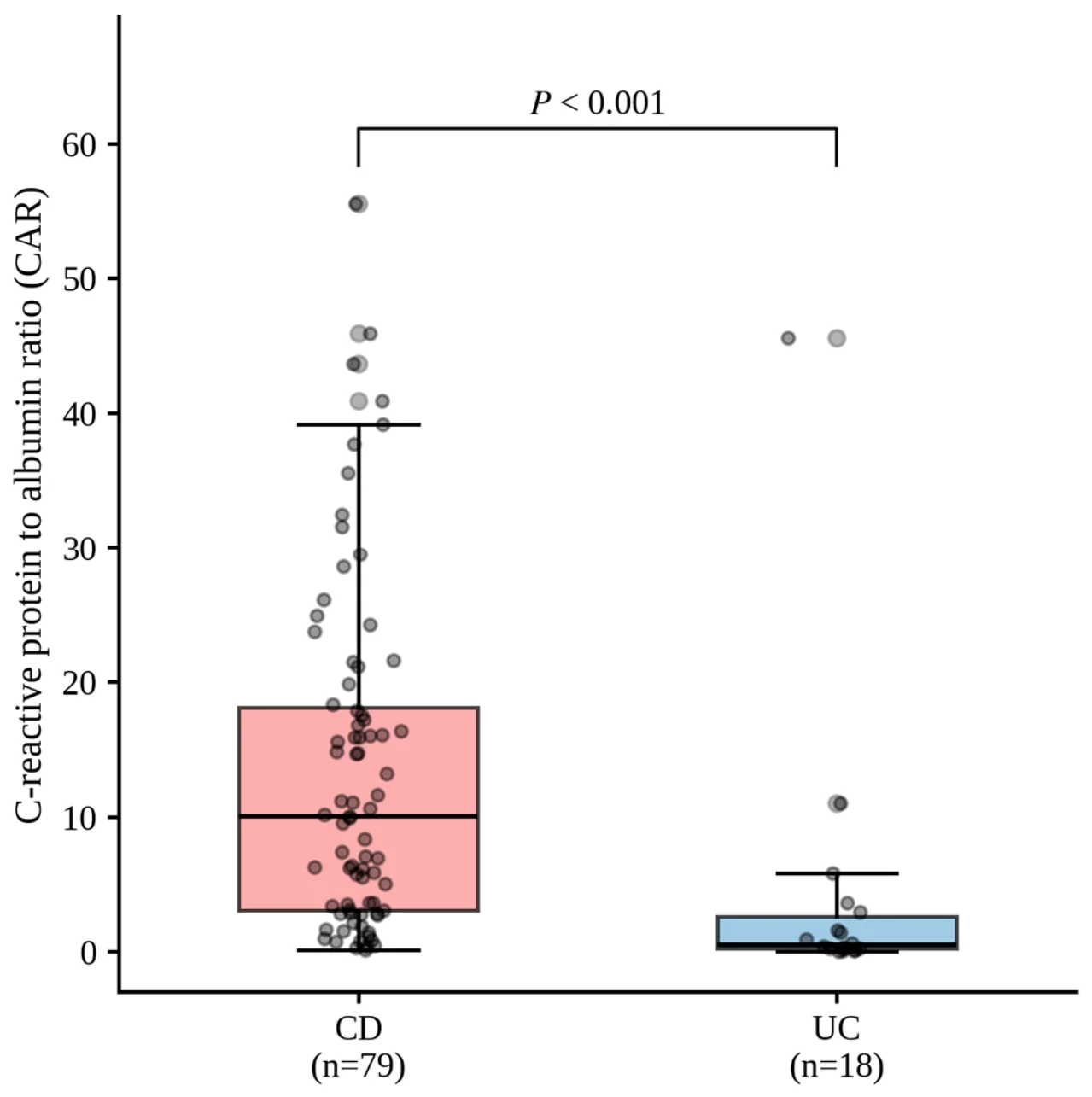
Comparison of the C-reactive protein-to-albumin ratio (CAR) between pediatric patients with Crohn’s disease (CD) and ulcerative colitis (UC). CAR was significantly higher in CD than UC (Mann–Whitney *U* test, *p* < 0.001). Box plots represent the median, interquartile range, and 1.5× interquartile range, with fill colour distinguishing CD (pink) from UC (blue); each grey dot represents one patient, and darker shading indicates overlapping points.

Regarding laboratory findings at diagnosis, hemoglobin was significantly lower in UC than CD (*p* = 0.003), while ESR, CRP, and CAR were significantly higher in CD than UC (all *p* < 0.001). Median CAR was 10.06 (range 0.10–55.55) in CD and 0.49 (range 0.02–45.55) in UC (Figure 2). WBC, platelet count, serum albumin, and fecal calprotectin did not differ significantly between groups. The reference standard classified every patient as either CD or UC, with no indeterminate result, and CRP and serum albumin—and therefore CAR—were available for all 97 patients; fecal calprotectin was available for 67 (55 CD, 12 UC), PCDAI for 64 of 79 CD patients, PUCAI for 16 of 18 UC patients, and the Mayo endoscopic subscore for 17 of 18 UC patients. No adverse events occurred in relation to venipuncture for the index test, and no serious adverse events related to sedation or endoscopy for the reference standard were recorded.

Most CD patients presented with ileocolonic involvement (L3, 82.1%), whereas UC patients showed varied disease extent. Regarding CD behavior, luminal disease (B1) was observed in 72 patients (91.1%), and complicated behavior (B2 or B3) in only 7 patients (8.9%). Perianal disease was present in 49 patients (62.0%) with CD. EEN and immunosuppressants were more frequently used in CD, while corticosteroids and 5-ASA were more frequently used in UC (all *p* < 0.05).

### 3.2. Correlation Between CAR and Clinical, Laboratory, and Endoscopic Parameters

Correlations between CAR and various clinical, laboratory, and endoscopic parameters in both groups are shown in Table 2. In CD, CAR showed significant positive correlations with platelet count (*r* = 0.396, *p* < 0.001), ESR (*r* = 0.474, *p* < 0.001), CRP (*r* = 0.990, *p* < 0.001), fecal calprotectin (*r* = 0.446, *p* = 0.001), PCDAI (*r* = 0.442, *p* < 0.001), and SES-CD (*r* = 0.446, *p* < 0.001). Significant negative correlations were observed with hemoglobin (*r* = −0.463, *p* < 0.001) and serum albumin (*r* = −0.643, *p* < 0.001).

Similar trends were observed in UC. CAR correlated positively with ESR (*r* = 0.568, *p* = 0.014), CRP (*r* = 0.979, *p* < 0.001), and Mayo endoscopic subscore (*r* = 0.493, *p* = 0.044), and negatively with hemoglobin (*r* = −0.668, *p* = 0.002) and serum albumin (*r* = −0.782, *p* < 0.001). In contrast to CD, the correlation between CAR and fecal calprotectin was not significant in UC (*r* = 0.336, *p* = 0.286), and the correlation between CAR and PUCAI did not reach significance (*r* = 0.421, *p* = 0.104). These UC analyses were based on 16 patients with an available PUCAI and 12 with an available fecal calprotectin measurement. At these sample sizes, correlations of the magnitude observed would not be expected to reach significance: detecting a correlation of *r* = 0.42 with 80% power at α = 0.05 requires approximately 42 patients, and detecting *r* = 0.34 requires approximately 67. The non-significant associations in UC are therefore uninformative regarding the presence or absence of a biological relationship.

### 3.3. CAR According to Disease Severity

CAR was compared across severity groups based on PCDAI and SES-CD in CD, and PUCAI and Mayo endoscopic subscore in UC.

**Figure 3:**
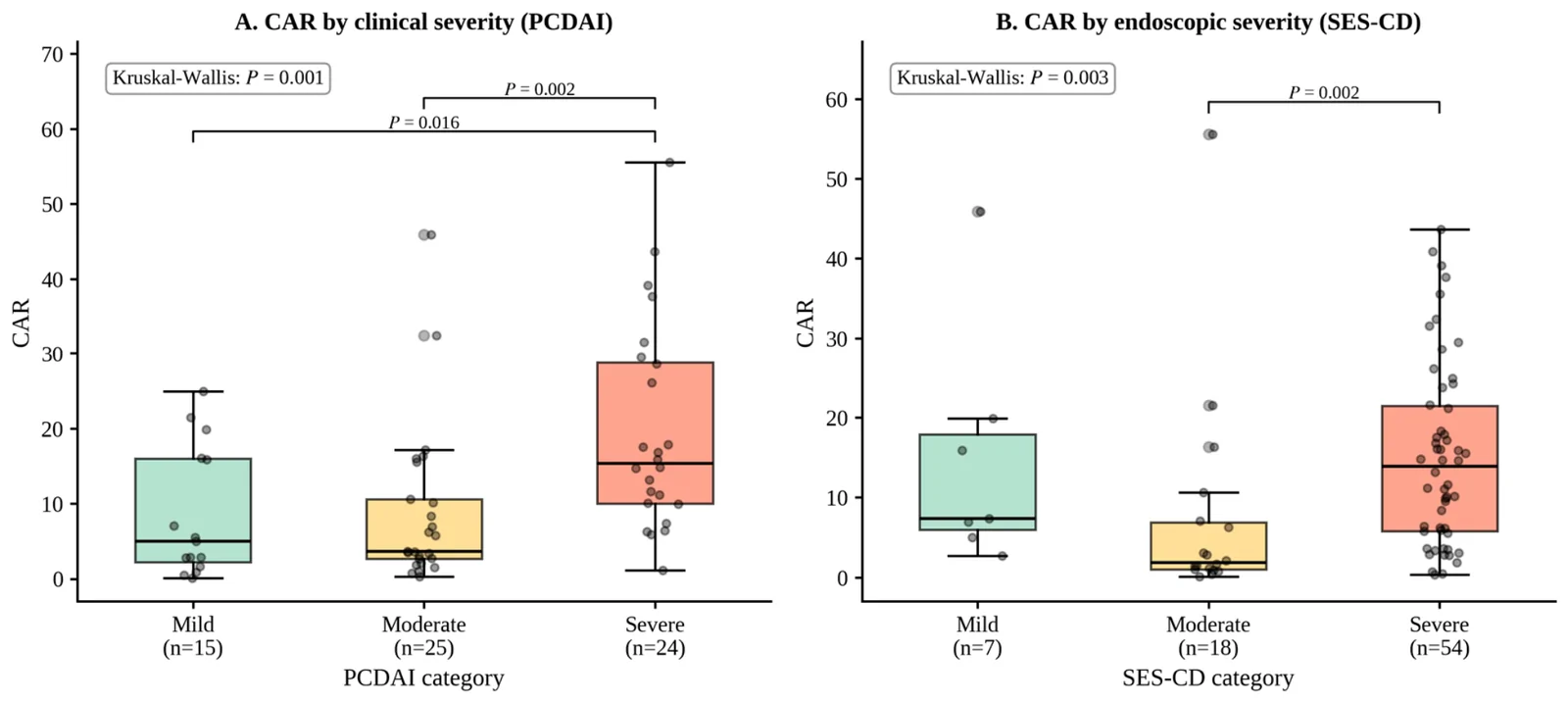
Comparison of CAR according to disease severity in CD. (**A**) CAR by clinical severity based on the Pediatric Crohn’s Disease Activity Index (PCDAI; *n* = 64). (**B**) CAR by endoscopic severity based on the Simple Endoscopic Score for Crohn’s Disease (SES-CD; *n* = 79). Statistical comparisons were performed using the Kruskal–Wallis test, with post hoc Mann–Whitney *U* tests using Bonferroni correction. Only *p*-values < 0.05 are shown. In both panels, box fill colour corresponds to the severity category labelled on the x-axis (green, mild; yellow, moderate; orange, severe); each grey dot represents one patient, and darker shading indicates overlapping points.

In CD, CAR differed significantly across PCDAI severity categories (Kruskal–Wallis *p* = 0.001) (Figure 3A). Median CAR was 5.01 (range 0.10–24.92) in mild (*n* = 15), 3.61 (range 0.31–45.92) in moderate (*n* = 25), and 15.35 (range 1.13–55.55) in severe (*n* = 24) groups. Post hoc analyses with Bonferroni correction showed that CAR in the severe group was significantly higher than in mild (*p* = 0.016) and moderate (*p* = 0.002) groups, with no difference between mild and moderate. CAR also differed across SES-CD severity categories (Kruskal–Wallis *p* = 0.003) (Figure 3B), with the severe group showing significantly higher CAR than the moderate group (*p* = 0.002).

In UC, no significant differences in CAR were observed across PUCAI severity categories (Kruskal–Wallis *p* = 0.079) or across Mayo endoscopic subscore categories (Kruskal–Wallis *p* = 0.141), although trends toward higher CAR in the severe group were noted. These findings may be attributable to the relatively small number of UC patients.

### 3.4. CAR According to Disease Behavior in CD

Subgroup analyses in CD evaluated the association between CAR and disease behavior. CAR was compared between CD patients with and without perianal disease, and between those with luminal (B1) and complicated (B2 or B3) behavior.

**Figure 4:**
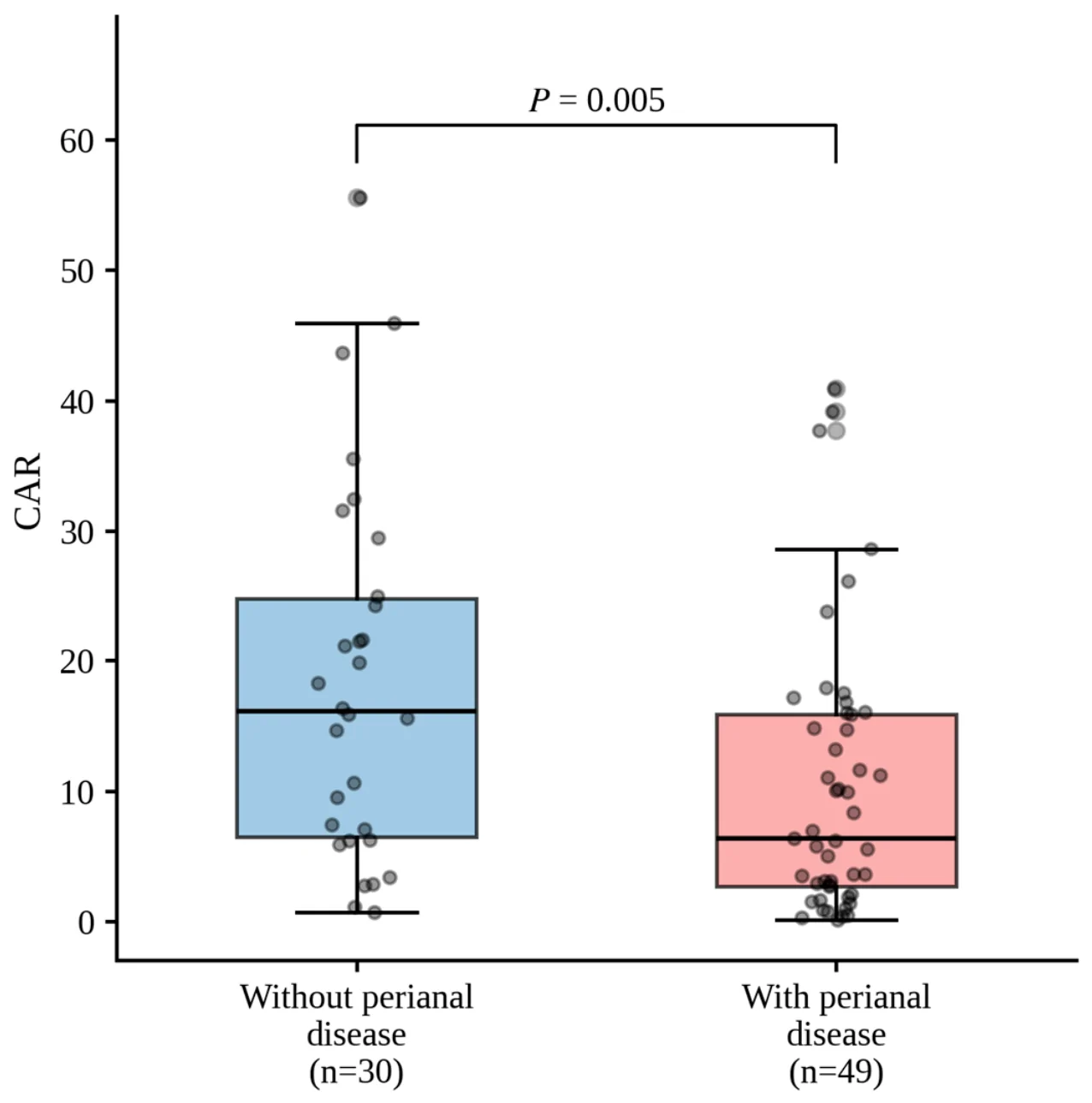
Comparison of CAR between CD patients with and without perianal disease. CAR was significantly higher in patients without perianal disease than in those with perianal disease (Mann–Whitney *U* test, *p* = 0.005). Box fill color corresponds to the group labelled on the x-axis (blue, without perianal disease; pink, with perianal disease); each grey dot represents one patient, and darker shading indicates overlapping points.

CAR was significantly higher in CD patients without perianal disease (*n* = 30, median 16.09, range 0.72–55.55) than in those with perianal disease (*n* = 49, median 6.39, range 0.10–40.89) (*p* = 0.005) (Figure 4). No significant difference in CAR was observed between B1 (*n* = 72, median 9.99) and B2/B3 (*n* = 7, median 10.62) (*p* = 0.560).

### 3.5. Diagnostic Performance of CAR for Distinguishing CD from UC

**Figure 5:**
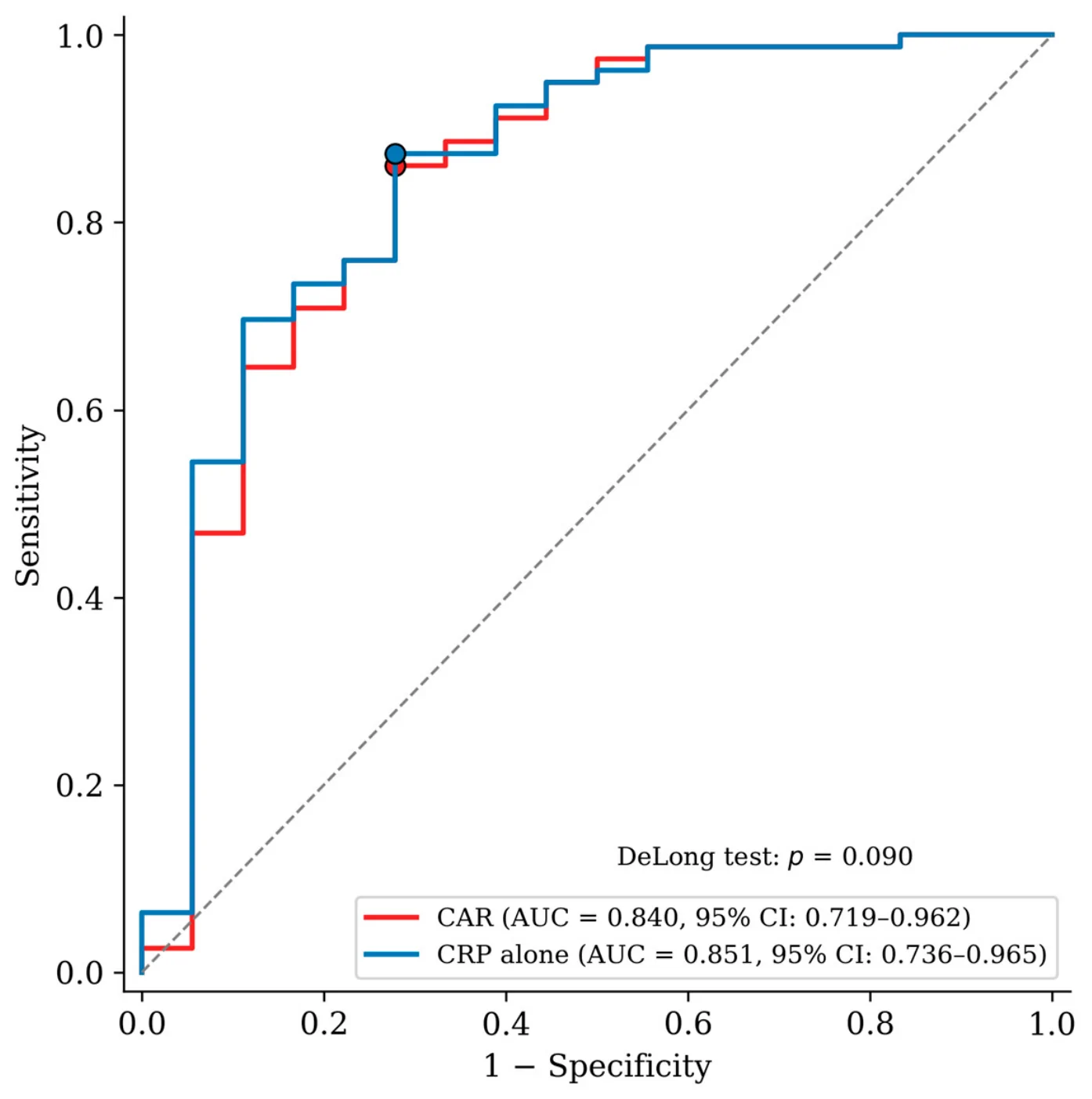
Receiver operating characteristic curves of CAR and of CRP alone for distinguishing CD from UC. The area under the curve was 0.840 (95% CI 0.719–0.962) for CAR and 0.851 (95% CI 0.736–0.965) for CRP alone; the two curves did not differ significantly (DeLong *p* = 0.090). Filled circles mark the cut-off maximizing the Youden index (CAR 1.62; CRP 6.5 mg/L). Curves and the DeLong comparison were computed with pROC.

The diagnostic performance of CAR was evaluated by ROC curve analysis (Figure 5). CAR discriminated CD from UC with an AUC of 0.840 (95% CI 0.719–0.962, DeLong method). The optimal cut-off was 1.62, yielding a sensitivity of 86.1% (95% CI 76.5–92.8) and a specificity of 72.2% (95% CI 46.5–90.3). The corresponding cross tabulation and accuracy measures are shown in Table 3. Positive predictive value was 93.2% (95% CI 84.7–97.7) and negative predictive value 54.2% (95% CI 32.8–74.4); these values reflect the 81.4% CD prevalence of this referral cohort and are not transferable to populations with a different case mix. The specificity estimate is imprecise, as it rests on only 18 UC patients.

Because CAR is a composite of CRP and albumin, we compared it with CRP alone. CRP by itself discriminated CD from UC at least as well as CAR (AUC 0.851, 95% CI 0.736–0.965), and the difference between the two curves was neither statistically significant nor in favor of CAR (difference in AUC −0.011, 95% CI −0.023 to +0.002; DeLong *p* = 0.090) (Figure 5, Table 3). Serum albumin alone discriminated poorly (AUC 0.612, 95% CI 0.447–0.777). CAR was almost perfectly correlated with CRP in both groups (*r* = 0.990 in CD and *r* = 0.979 in UC; Table 2), indicating that the ratio is dominated by its numerator in this cohort and that dividing by albumin did not add discriminative information.

**Table 3:** Diagnostic performance of the C-reactive protein-to-albumin ratio (CAR) and of C-reactive protein (CRP) alone for distinguishing pediatric Crohn’s disease from ulcerative colitis. (a) Cross tabulation of CAR against the reference standard at the 1.62 cut-off; (b) measures of diagnostic accuracy (95% confidence intervals).

(**a**)			
	**CD (** ***n*** ** = 79)**	**UC (** ***n*** ** = 18)**	**Total**
CAR ≥ 1.62	68	5	73
CAR < 1.62	11	13	24
Total	79	18	97
(**b**)			
**Measure**	**CAR**		**CRP Alone**
AUC	0.840 (0.719–0.962)		0.851 (0.736–0.965)
Optimal cut-off (Youden)	1.62		6.5 mg/L
Sensitivity, %	86.1 (76.5–92.8)		87.3 (78.0–93.8)
Specificity, %	72.2 (46.5–90.3)		72.2 (46.5–90.3)
Positive predictive value, %	93.2 (84.7–97.7)		93.2 (84.9–97.8)
Negative predictive value, %	54.2 (32.8–74.4)		56.5 (34.5–76.8)
Accuracy, %	83.5 (74.6–90.3)		84.5 (75.8–91.1)
Positive likelihood ratio	3.10		3.14
Negative likelihood ratio	0.19		0.18

The target condition is Crohn’s disease; ulcerative colitis represents the alternative diagnosis. Cut-offs were derived from these data by maximizing the Youden index and are therefore exploratory rather than pre-specified. AUCs are reported with DeLong 95% confidence intervals; sensitivity, specificity, predictive values, and accuracy with exact (Clopper–Pearson) binomial 95% confidence intervals. The difference in AUC between CAR and CRP alone was −0.011 (95% CI −0.023 to +0.002; DeLong *p* = 0.090). Predictive values reflect the 81.4% prevalence of Crohn’s disease in this referral cohort. AUC, area under the receiver operating characteristic curve; CAR, C-reactive protein-to-albumin ratio; CD, Crohn’s disease; CRP, C-reactive protein; UC, ulcerative colitis.

### 3.6. CAR During One-Year Follow-Up

The clinical course was followed for one year after IBD diagnosis. Among the 97 patients, 16 patients (15 with follow-up loss and 1 without available data) were excluded from the relapse analysis. Baseline comparison between the 16 excluded patients and the 81 included patients revealed no statistically significant differences in initial CAR (median 7.70 vs. 6.18, *p* = 0.988), age at diagnosis (*p* = 0.490), sex distribution (*p* = 0.780), CD-to-UC ratio (*p* = 0.168), or other major laboratory and clinical parameters at diagnosis (all *p* > 0.05), suggesting that selection bias due to loss to follow-up was likely minimal. Of the remaining 81 patients, 51 (63.0%) maintained remission and 30 (37.0%) experienced relapse during the one-year follow-up.

Initial CAR at diagnosis did not significantly differ between the remission group (median 5.89, range 0.02–45.55) and the relapse group (median 8.72, range 0.23–55.55) (*p* = 0.509). At one-year follow-up, CAR tended to be higher in the relapse group (median 0.747, range 0.016–30.932) than in the remission group (median 0.156, range 0.026–8.824), although the difference did not reach significance (*p* = 0.055).

**Figure 6:**
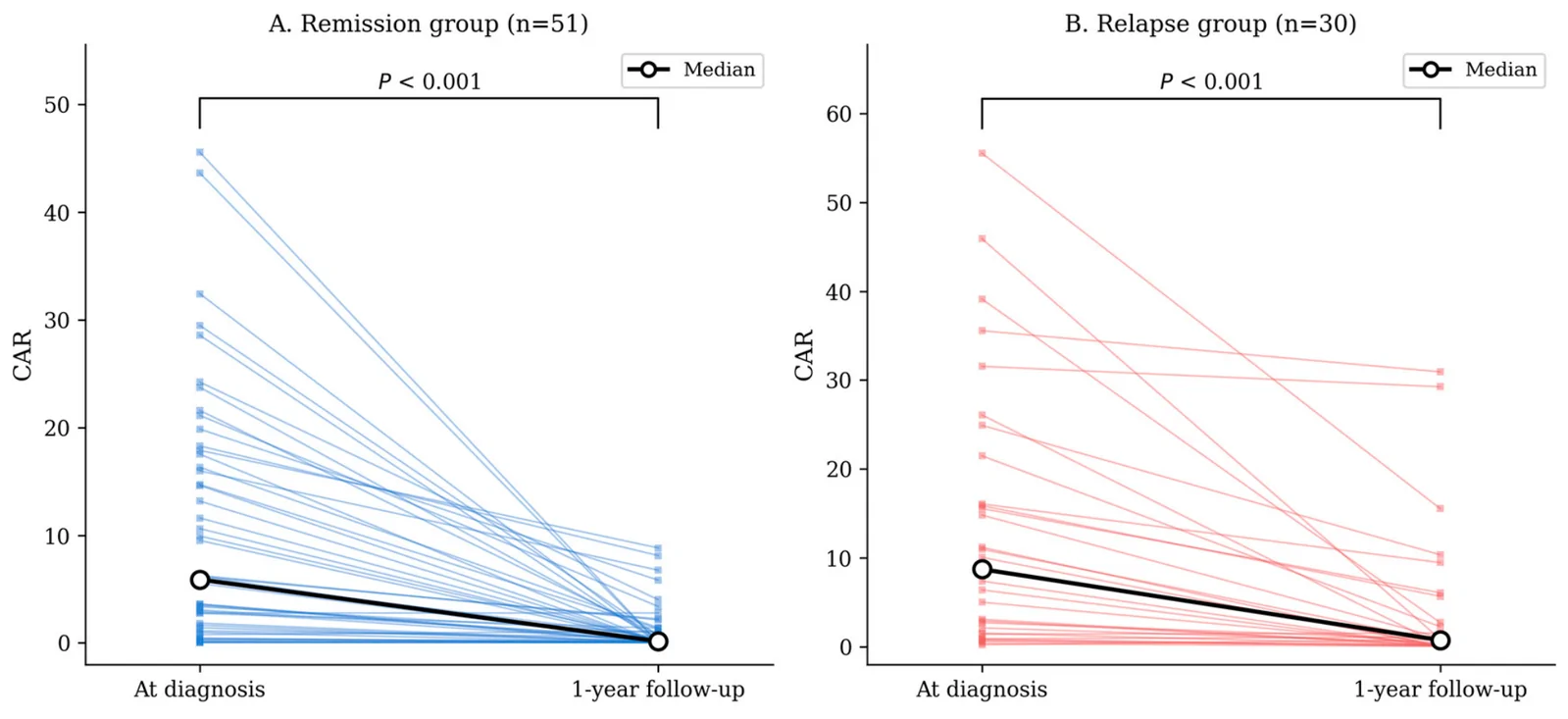
Changes in CAR from diagnosis to one-year follow-up, stratified by clinical outcome at one year. (**A**) Remission group (*n* = 51). (**B**) Relapse group (*n* = 30). In both groups, CAR significantly decreased over time (Wilcoxon signed-rank test, *p* < 0.001 for both). Thin colored lines represent individual patients; thick black lines connect group medians.

Paired analyses revealed that CAR significantly decreased from diagnosis to one-year follow-up in both the remission group (initial median 5.89 vs. 1-year median 0.156, Wilcoxon *p* < 0.001) and the relapse group (initial median 8.72 vs. 1-year median 0.747, Wilcoxon *p* < 0.001) (Figure 6). Nevertheless, CAR in the relapse group remained relatively higher at one-year follow-up than in the remission group.

## 4. Discussion

The present study investigated the role of CAR as a non-invasive biomarker reflecting disease severity, behavior, and clinical course in pediatric patients with IBD, focusing on the comparison between CD and UC. CAR was significantly higher in CD than UC and discriminated between them with an AUC of 0.840, but it did not outperform CRP alone (AUC 0.851; DeLong *p* = 0.090), and the point estimate in fact favored CRP. CAR correlated significantly with established inflammatory markers and disease activity indices in both diseases, although these correlations were stronger and more consistent in CD. CAR differed across disease severity categories in CD but not in UC, and significantly decreased from diagnosis to one-year follow-up regardless of subsequent clinical course. Unexpectedly, CAR was higher in CD patients without perianal involvement than in those with perianal disease, a finding that may reflect distinct phenotypic characteristics of perianal CD.

CAR, calculated as the ratio of serum CRP to albumin, reflects both systemic inflammation and nutritional status. Elevated CRP and decreased albumin levels both reflect ongoing inflammation, as CRP is a positive acute-phase protein synthesized by the liver in response to interleukin-6, while albumin is a negative acute-phase protein whose synthesis is downregulated during systemic inflammation. In adult IBD, several studies have reported the clinical utility of CAR in monitoring disease activity, predicting treatment response, and assessing prognosis. Qin et al. demonstrated that CAR was a useful biomarker for assessing disease activity in adult CD [11], while subsequent studies have validated CAR as a prognostic marker in both CD and UC [12,13,21]. A recent systematic review and meta-analysis by Li et al. further confirmed that immune-inflammation indices, including CAR, are significantly associated with disease severity and adverse clinical outcomes in IBD [22]. Data on CAR in pediatric IBD remain limited, with only a single multi-center study by Glapa-Nowak et al. reporting the diagnostic utility of CAR in 345 pediatric IBD patients [14]. To our knowledge, the present study is among the first to systematically evaluate CAR not only as a diagnostic marker but also in association with disease behavior and clinical course in pediatric IBD.

In the present study, CAR was significantly higher in CD than UC, with markedly different distributions. This finding is consistent with prior reports that CRP elevation is more prominent in CD than UC [9,23], which may be attributable to the transmural nature of CD inflammation in contrast to the mucosal inflammation of UC. Approximately 15–20% of pediatric UC patients with moderate disease activity have been reported to exhibit normal CRP levels, particularly when inflammation is limited to the rectum or sigmoid colon [24,25]. The diagnostic performance of CAR for distinguishing CD from UC (AUC 0.840, sensitivity 86.1%, specificity 72.2% at a cut-off of 1.62) was, however, no better than that of CRP measured alone (AUC 0.851). This is the central negative finding of our study, and it has a straightforward explanation: serum albumin varied over a narrow range in this cohort and discriminated poorly on its own (AUC 0.612), so dividing CRP by albumin produced an index almost perfectly correlated with its numerator (*r* = 0.990 in CD) without adding information. Several adult IBD studies have compared CAR with CRP alone in the context of assessing disease activity [11,12,13,21], whereas the only prior pediatric report did not include such a benchmark [14]. To our knowledge, however, no previous study in either adult or pediatric populations has formally tested whether CAR outperforms CRP alone for discriminating CD from UC. The implication is not that CAR is uninformative but that its apparent diagnostic value should not be attributed to the ratio itself. Where CRP is available—which is wherever CAR can be calculated, since CRP is its numerator—the simpler measure performed at least as well. Definitive diagnosis in any case requires comprehensive evaluation including endoscopy and histopathology, and neither marker is a substitute for it.

The absence of a diagnostic advantage does not exclude a role for CAR during follow-up. CRP reflects the acute-phase response alone, whereas the albumin denominator introduces a slower-moving term influenced by enteric protein loss, hepatic reprioritization of synthesis, and nutritional intake [26,27]. These are dimensions of disease burden that are clinically prominent in children, in whom restored growth and nutritional recovery are treatment targets in their own right alongside biomarker normalization [7,8]. In our cohort, albumin varied over a narrow range at diagnosis and discriminated poorly on its own (AUC 0.612), which is consistent with the ratio contributing little beyond CRP at a single cross-sectional time point. Any incremental contribution would instead be expected to emerge across serial measurements during treatment, when systemic inflammation and nutritional status recover on different timescales—a possibility our one-year data are suggestive of but not powered to test. We regard this as a hypothesis generated by the present study rather than one that it establishes.

CAR showed significant positive correlations with clinical and endoscopic activity scores in CD and differed significantly across disease severity categories. The severe CD group exhibited markedly higher CAR than the mild and moderate groups by PCDAI, and higher CAR than the moderate group by SES-CD. These findings extend prior reports demonstrating that CAR reflects disease activity in adult CD [11,12,13] to the pediatric population. The magnitude of these associations warrants attention alongside their statistical significance. The correlations observed in CD between CAR and clinical or endoscopic activity (*r* = 0.442 and *r* = 0.446, respectively) correspond to approximately 20% of shared variance, indicating a moderate group-level association rather than a basis for individual-level prediction. An independent adult cohort that benchmarked several composite inflammatory indices against endoscopic and histological activity reported similarly moderate accuracy for CAR (AUC 0.682) [28]. The near-perfect correlation between CAR and CRP in our cohort (*r* = 0.990) should likewise be understood as an arithmetic consequence of how the ratio is constructed rather than as evidence of biological convergence. We therefore position CAR as a supportive marker of disease activity at the group level, not as a substitute for endoscopic assessment in the individual patient. In UC, although a trend toward higher CAR in the severe group was observed, differences across severity categories did not reach significance. This may reflect the relatively small UC sample size and the well-documented phenomenon that CRP elevation is less consistent in UC than CD, particularly in pediatric patients [23,24,25]. With only 18 UC patients, however, these analyses had insufficient power to detect associations of the magnitude seen in CD, and they should not be read as evidence that no such association exists; the correlation between CAR and the Mayo endoscopic subscore did reach significance in this subgroup (*r* = 0.493, *p* = 0.044). Whether the utility of CAR genuinely differs between pediatric CD and UC therefore remains an open question.

An unexpected finding was that CAR was significantly higher in CD patients without perianal involvement than in those with perianal disease. This contrasts with the general perception that perianal CD represents a more aggressive phenotype [29,30,31]. Two explanations may account for this observation. First, perianal manifestations such as fissures, fistulas, or skin tags often prompt earlier medical evaluation, leading to diagnosis before extensive luminal inflammation develops. Second, perianal CD may represent a phenotypically distinct subtype in which the inflammatory process is localized to the perianal region, eliciting a less prominent systemic acute-phase response. No significant difference in CAR was observed between B1 and B2/B3 behavior, likely owing to the small number of patients with complicated behavior (*n* = 7). Together, these findings suggest that CAR may primarily reflect the extent of luminal inflammation rather than perianal involvement per se, warranting further studies with larger cohorts.

Regarding one-year follow-up, CAR significantly decreased from diagnosis in both the remission and relapse groups, indicating that CAR responds to treatment in pediatric IBD. However, initial CAR at diagnosis did not significantly differ between patients who maintained remission and those who relapsed, suggesting that a single measurement at diagnosis may not suffice to predict the long-term course. CAR at the one-year follow-up tended to be higher in the relapse group than the remission group, although this difference did not reach significance. These findings imply that serial CAR measurements during follow-up may be more informative than a single baseline measurement for monitoring disease activity and predicting clinical outcomes. Supporting this notion, Zhang et al. recently reported that CAR was useful for predicting treatment response and long-term clinical remission following infliximab therapy in adult UC [32], underscoring the potential value of serial CAR measurements during treatment in IBD.

Fecal calprotectin is currently considered the most reliable non-invasive biomarker for detecting intestinal inflammation in pediatric IBD [33,34]. However, fecal calprotectin requires stool sampling, has limited availability in some settings, and is relatively expensive. In the present study, CAR showed a significant positive correlation with fecal calprotectin in CD, supporting CAR’s validity as a marker of intestinal inflammation [33]. Although fecal calprotectin remains an important biomarker, a serum acute-phase measure may serve as a complementary, readily available, and cost-effective adjunct when stool sampling is not feasible or rapid assessment is needed. Our comparison with CRP alone indicates, however, that this role does not require the ratio: CRP by itself carries essentially the same information in this cohort, and any advantage claimed for CAR over CRP would need to be demonstrated rather than assumed.

The present study has several limitations. First, this was a retrospective single-center study, and the UC group comprised only 18 patients. This limited the power to detect differences across UC severity categories, left the specificity estimate very imprecise (95% CI 46.5–90.3), and left the comparison of CAR with CRP alone underpowered; a difference in AUC favoring CRP cannot be excluded, nor can a small advantage for CAR, and the *p* = 0.090 should be read as inconclusive rather than as evidence of equivalence. Second, the cut-off of 1.62 was derived from these same data by maximizing the Youden index and was not pre-specified, so the reported sensitivity and specificity are optimistic and require external validation. Third, fecal calprotectin was available in only 67 of 97 patients and clinical activity indices in 80 of 97, and analyses were complete-case, which may have introduced selection bias. Fourth, although the difference in age at diagnosis between the CD and UC groups did not reach statistical significance (*p* = 0.052), this borderline difference may have acted as a residual confounder; further studies with age-matched cohorts or multivariate adjustment are warranted. Fifth, the follow-up period was limited to one year; longer-term observation is needed to fully evaluate the prognostic value of CAR in pediatric IBD. Lastly, the definition of relapse was based on the necessity of treatment escalation, which is clinically meaningful but not standardized, and predictive values are conditioned on the 81.4% CD prevalence of a tertiary referral cohort and would be substantially lower in a primary-care setting. Our analyses were also univariate throughout. We considered multivariable models adjusting for age, sex, hemoglobin, ESR, fecal calprotectin, disease location, and nutritional status, but did not fit them, for two reasons. First, the number of events available—24 patients with severe disease by PCDAI among the 64 CD patients with an available score—supports approximately two covariates at the conventional threshold of ten events per variable, whereas the covariate set above requires approximately ten parameters once disease location is entered categorically. Second, CAR is arithmetically derived from CRP and albumin and was almost perfectly correlated with CRP in this cohort (*r* = 0.990), so that entering CRP, albumin, or ESR alongside CAR would partition variance within a single quantity rather than adjust for confounding. We therefore report our findings as associations and do not claim that CAR contributes information independently of established inflammatory markers; establishing independence will require a cohort with enough events to support an adequately specified model, as has been achieved for other composite serum ratios in larger IBD populations [35]. For the same reason we did not formally model relapse: 30 of the 81 patients with complete one-year follow-up (37.0%) relapsed, which supports approximately three covariates, whereas a clinically meaningful prognostic model would need to include baseline CAR, diagnosis, baseline disease activity, and treatment received during follow-up. Time-to-event analysis was not possible because our records documented whether relapse occurred within the follow-up year but not a reliable date of onset. The longitudinal findings reported here are therefore descriptive, and the present study should be taken as evidence neither for nor against a prognostic role for baseline CAR. Finally, patients were recruited across a twelve-year interval (2012–2023) during which pediatric IBD management changed substantially, with earlier and more frequent initiation of biologic therapy both internationally [7] and in Korea, where nationwide data document a marked expansion in anti-tumor necrosis factor prescribing over this period [4]. Only 9 of 79 CD patients (11.7%) and 1 of 18 UC patients (5.6%) in our cohort received biologics, proportions that reflect practice averaged across the recruitment window rather than contemporary practice; because treatment intensity influences both the trajectory of inflammatory markers and the probability of meeting our relapse definition, secular change over the recruitment period may have contributed to the one-year outcomes reported here. Future prospective, multi-center studies with larger cohorts and longer follow-up are warranted to validate the clinical utility of CAR in pediatric IBD.

## 5. Conclusions

CAR is a readily available, low-cost marker that correlates with clinical and endoscopic disease activity in pediatric CD, differentiates severe from milder disease, and falls significantly after treatment. It did not, however, discriminate CD from UC any better than CRP measured alone, and its association with activity should therefore be understood as largely inherited from its CRP component rather than as a property of the ratio. On present evidence, CAR cannot be recommended in preference to CRP for distinguishing CD from UC in children. Its plausible role is in longitudinal activity assessment, where the albumin term may capture the nutritional dimension of disease burden that CRP alone does not; testing that hypothesis will require prospective cohorts large enough to compare CAR against CRP directly, with albumin varying over a wider range than in the present sample.

## Figures and Tables

**Table 1:** Comparison of demographic features, laboratory findings, disease activity, and treatment between pediatric Crohn’s disease and ulcerative colitis.

Variable	CD (*n* = 79)	UC (*n* = 18)	*p*-Value
Male, *n* (%)	57 (72.2)	5 (27.8)	0.001
Age at diagnosis, years	14.8 (9.1–18.5)	12.9 (8.2–17.7)	0.052
Z-score of height	1.25 (−4.11 to 5.73)	1.20 (−3.41 to 4.34)	0.738
Z-score of weight	−0.08 (−3.89 to 3.56)	−0.08 (−3.48 to 2.70)	0.827
Z-score of BMI	−0.58 (−4.25 to 2.05)	−0.24 (−2.24 to 1.52)	0.577
*Laboratory findings*			
WBC (/μL)	9620 (4130–17,850)	8155 (3990–17,760)	0.141
Hemoglobin (g/dL)	12.20 (7.20–16.70)	10.50 (7.20–14.80)	0.003
Platelet (×10^3^/μL)	468 (253–797)	398 (191–708)	0.438
ESR (mm/h)	60 (10–120)	20 (2–102)	<0.001
CRP (mg/L)	36.51 (0.44–177.77)	2.19 (0.11–118.44)	<0.001
Serum albumin (g/dL)	3.80 (2.50–5.30)	4.10 (2.60–5.10)	0.141
CAR	10.06 (0.10–55.55)	0.49 (0.02–45.55)	<0.001
Fecal calprotectin (mg/kg)	929 (26–13,407)	2061 (159–13,409)	0.199
*Clinical disease activity*			
PCDAI/PUCAI	35 (5–75)	50 (25–70)	–
Mild, *n* (%)	15 (23.4)	4 (25.0)	–
Moderate, *n* (%)	25 (39.1)	10 (62.5)	–
Severe, *n* (%)	24 (37.5)	2 (12.5)	–
*Endoscopic disease activity*			
SES-CD/Mayo	21 (4–34)	2 (1–3)	–
Mild, *n* (%)	7 (8.9)	2 (11.8)	–
Moderate, *n* (%)	18 (22.8)	7 (41.2)	–
Severe, *n* (%)	54 (68.4)	8 (47.1)	–
*Disease location, n (%)*			
L1/E1	6 (7.7)	4 (23.5)	–
L2/E2	7 (9.0)	6 (35.3)	–
L3/E3	64 (82.1)	4 (23.5)	–
L4/E4	1 (1.3)	3 (17.6)	–
*CD behavior, n (%)*			
B1 (luminal)	72 (91.1)	–	–
B2 or B3 (complicated)	7 (8.9)	–	–
Perianal disease	49 (62.0)	–	–
*Treatment, n (%)*			
EEN	59 (75.6)	0 (0.0)	<0.001
Corticosteroid	21 (26.9)	15 (83.3)	<0.001
5-ASA	5 (6.4)	6 (33.3)	0.005
Immunosuppressant	73 (93.6)	12 (66.7)	0.005
Biologics	9 (11.7)	1 (5.6)	0.681

Data are presented as numbers (%) for categorical variables or median (range) for continuous variables. Continuous variables were compared using the Mann–Whitney *U* test; categorical variables were compared using the chi-squared test or Fisher’s exact test, as appropriate. *p*-Values < 0.05 were considered statistically significant. Denominators differ between rows where data were incomplete: z-scores were available for 78 CD and 17 UC patients; disease location for 78 CD and 17 UC; fecal calprotectin for 55 CD and 12 UC; PCDAI for 64 CD; PUCAI for 16 UC; the Mayo endoscopic subscore for 17 UC; treatment data for 78 CD (77 for biologics). Percentages are calculated on the number with available data. 5-ASA, 5-aminosalicylic acid; BMI, body mass index; CAR, C-reactive protein-to-albumin ratio; CD, Crohn’s disease; CRP, C-reactive protein; EEN, exclusive enteral nutrition; ESR, erythrocyte sedimentation rate; PCDAI, Pediatric Crohn’s Disease Activity Index; PUCAI, Pediatric Ulcerative Colitis Activity Index; SES-CD, Simple Endoscopic Score for Crohn’s Disease; UC, ulcerative colitis; WBC, white blood cell.

**Table 2:** Correlation between the C-reactive protein-to-albumin ratio (CAR) and other clinical, laboratory, and endoscopic parameters in pediatric inflammatory bowel disease.

Variable	*r* (CD)	*p*-Value (CD)	*r* (UC)	*p*-Value (UC)
WBC	0.110	0.336	0.366	0.135
Hemoglobin	−0.463	<0.001	−0.668	0.002
Platelet	0.396	<0.001	0.424	0.079
ESR	0.474	<0.001	0.568	0.014
CRP	0.990	<0.001	0.979	<0.001
Serum albumin	−0.643	<0.001	−0.782	<0.001
Fecal calprotectin	0.446	0.001	0.336	0.286
PCDAI/PUCAI	0.442	<0.001	0.421	0.104
SES-CD/Mayo	0.446	<0.001	0.493	0.044

Correlation analyses were performed using Spearman’s rank correlation and are complete-case: *n* = 79 for CD and 18 for UC except fecal calprotectin (55 CD, 12 UC), PCDAI (64 CD), PUCAI (16 UC), and Mayo (17 UC). *p*-Values < 0.05 were considered statistically significant. CAR, C-reactive protein-to-albumin ratio; CD, Crohn’s disease; CRP, C-reactive protein; ESR, erythrocyte sedimentation rate; PCDAI, Pediatric Crohn’s Disease Activity Index; PUCAI, Pediatric Ulcerative Colitis Activity Index; SES-CD, Simple Endoscopic Score for Crohn’s Disease; UC, ulcerative colitis; WBC, white blood cell.

## Data Availability

The data presented in this study are available on request from the corresponding author. The data are not publicly available due to privacy and ethical restrictions concerning pediatric patient information.

## Supplementary Materials

The following supporting information can be downloaded at: https://www.mdpi.com/article/10.3390/diagnostics16162608/s1, STARD 2015 Checklist. Reference [36] is cited in the supplementary materials.

## Abbreviations

The following abbreviations are used in this manuscript:

5-ASA	5-aminosalicylic acid
AUC	Area under the curve
BMI	Body mass index
CAR	C-reactive protein-to-albumin ratio
CD	Crohn’s disease
CI	Confidence interval
CRP	C-reactive protein
EEN	Exclusive enteral nutrition
ESR	Erythrocyte sedimentation rate
IBD	Inflammatory bowel disease
PCDAI	Pediatric Crohn’s Disease Activity Index
PUCAI	Pediatric Ulcerative Colitis Activity Index
ROC	Receiver operating characteristic
SES-CD	Simple Endoscopic Score for Crohn’s Disease
STROBE	Strengthening the Reporting of Observational Studies in Epidemiology
UC	Ulcerative colitis
WBC	White blood cell
