# Supplementary material for: C-Reactive Protein-to-Albumin Ratio in Pediatric Inflammatory Bowel Disease: Associations with Disease Activity and Comparison with C-Reactive Protein Alone
Source: Diagnostics (Basel). 2026 Aug 17;16(16):2608. doi: 10.3390/diagnostics16162608 (PMC13511885; doi:10.3390/diagnostics16162608)
Supplement: Supplementary file 1 [file diagnostics-16-02608-s001.zip › diagnostics-4473356-supplementary.pdf]

STARD 2015 Checklist

Manuscript title: C-Reactive Protein-to-Albumin Ratio as a Non-Invasive Biomarker for Disease Severity and Behavior in Pediatric Inflammatory Bowel Disease

Journal: *Diagnostics* (MDPI) — Section: Clinical Diagnosis and Prognosis

**Index test**	C-reactive protein-to-albumin ratio (CAR) at diagnosis
**Reference standard**	Diagnosis and classification of Crohn's disease vs. ulcerative colitis according to the revised ESPGHAN Porto criteria (clinical, laboratory, endoscopic with histopathology, and radiologic evaluation)
**Target condition**	Crohn's disease (vs. ulcerative colitis) in pediatric inflammatory bowel disease

Section numbers refer to the revised manuscript. All 30 items are addressed; no item remains open.

Section & Topic	No.	Item	Reported in
**TITLE OR ABSTRACT**			
	1	Identification as a study of diagnostic accuracy using at least one measure of accuracy (such as sensitivity, specificity, predictive values, or AUC)	Title; Abstract
**ABSTRACT**			
	2	Structured summary of study design, methods, results, and conclusions	Abstract
**INTRODUCTION**			
	3	Scientific and clinical background, including the intended use and clinical role of the index test	Section 1, paragraphs 1–4
	4	Study objectives and hypotheses	Section 1, paragraph 5
**METHODS**			
Study design	5	Whether data collection was planned before the index test and reference standard were performed (prospective study) or after (retrospective study)	Section 2.1 (retrospective)
Participants	6	Eligibility criteria	Section 2.1
	7	On what basis potentially eligible participants were identified	Section 2.1

Section & Topic	No.	Item	Reported in
	8	Where and when potentially eligible participants were identified (setting, location and dates)	Section 2.1
	9	Whether participants formed a consecutive, random or convenience series	Section 2.1 (consecutive)
Test methods	10a	Index test, in sufficient detail to allow replication	Section 2.3
	10b	Reference standard, in sufficient detail to allow replication	Sections 2.1, 2.4
	11	Rationale for choosing the reference standard (if alternatives exist)	Sections 1, 2.1
	12a	Definition of and rationale for test positivity cut-offs or result categories of the index test, distinguishing pre-specified from exploratory	Sections 2.6, 3.5 — cut-off 1.62 derived by maximum Youden index; **exploratory, not pre-specified**
	12b	Definition of and rationale for test positivity cut-offs or result categories of the reference standard, distinguishing pre-specified from exploratory	Sections 2.1, 2.4 — pre-specified (revised Porto criteria; Paris classification)
	13a	Whether clinical information and reference standard results were available to the performers/readers of the index test	Section 2.3
	13b	Whether clinical information and index test results were available to the assessors of the reference standard	Section 2.3
Analysis	14	Methods for estimating or comparing measures of diagnostic accuracy	Section 2.6 (ROC, Youden index, DeLong test, Clopper–Pearson CIs; R 4.3.3 with pROC 1.18.5)
	15	How indeterminate index test or reference standard results were handled	Section 3.1 (no indeterminate results occurred; the reference standard classified every

Section & Topic	No.	Item	Reported in
			patient as CD or UC)
	16	How missing data on the index test and reference standard were handled	Section 2.1; Section 2.6 (complete-case, no imputation); Section 3.1 (availability by variable); footnotes to Tables 1 and 2
	17	Any analyses of variability in diagnostic accuracy, distinguishing pre-specified from exploratory	Sections 3.3, 3.4 — exploratory subgroup analyses
	18	Intended sample size and how it was determined	Section 2.1 (no a priori calculation; size of available cohort)
**RESULTS**			
Participants	19	Flow of participants, using a diagram	Figure 1
	20	Baseline demographic and clinical characteristics of participants	Section 3.1; Table 1
	21a	Distribution of severity of disease in those with the target condition	Section 3.1; Table 1 (PCDAI, SES-CD, Paris behavior and location for CD)
	21b	Distribution of alternative diagnoses in those without the target condition	Section 3.1; Table 1 (PUCAI, Mayo subscore, disease extent for UC)
	22	Time interval and any clinical interventions between index test and reference standard	Section 2.3 (same visit; no intervening treatment)
Test results	23	Cross tabulation of the index test results (or their distribution) by the results of the reference standard	Section 3.5; Table 3(a); Figures 1, 2
	24	Estimates of diagnostic accuracy and their precision (such as 95% confidence intervals)	Section 3.5; Table 3(b); Figure 5
	25	Any adverse events from performing the index test or the reference standard	Section 3.1
**DISCUSSION**			
	26	Study limitations, including sources of potential bias, statistical uncertainty, and generalisability	Section 4, final paragraph (incl. data-derived cut-off, imprecise specificity,

Section & Topic	No.	Item	Reported in
			prevalence-dependent predictive values)
	27	Implications for practice, including the intended use and clinical role of the index test	Sections 4, 5
**OTHER INFORMATION**			
	28	Registration number and name of registry	Not applicable — retrospective observational study, not registered
	29	Where the full study protocol can be accessed	Not applicable — no publicly accessible protocol
	30	Sources of funding and other support; role of funders	Funding statement

Notes

All 30 STARD items are addressed. Items 9, 13a, 13b, 14, 16, 23, 24, 25 and 26 were completed from the source dataset (CRF_renewal.xlsx); items 18, 19 and 22 from author confirmation.

Item 19 (flow diagram). No patient met an exclusion criterion, so all 97 consecutive patients assessed for eligibility were analyzed. Figure 1 records this explicitly (Excluded, n = 0) rather than omitting the step, since a reviewer needs to see that screening was considered.

Item 22 (interval between index test and reference standard). CRP and albumin were drawn at the same visit as the diagnostic endoscopy, with no intervening treatment. This is the strongest available answer: a zero interval removes disease-progression bias, and the absence of prior corticosteroids or exclusive enteral nutrition removes the main source of index-test bias in this setting. Stated in Section 2.3 and in the Figure 1 caption.

Software (item 14). All analyses were run in SPSS 22.0 except the diagnostic accuracy analysis, which was run in R 4.3.3 with pROC 1.18.5 so that Table 3 and Figure 5 derive from a single method. This analysis has been executed and its output is provided (`Diagnostics_ROC_output.txt`, reproducible via `roc_analysis.R`); every value in Table 3 and Section 3.5 matches that output. The cut-off is reported as 1.62 because pROC returns the midpoint between adjacent observed values; this defines the same classification rule as the observed value 1.64 and yields an identical 2 × 2 table.

Data cleaning. One Mayo endoscopic subscore entered in the case report form as 4 — outside the 0–3 range of the instrument — was verified against the endoscopy report and corrected to 3 before analysis. This is documented in Section 2.4. Retain that sentence: the CAR–Mayo correlation is $r = 0.493$, $p = 0.044$ with the correction and $p = 0.052$ without it,

so the disclosure is what makes a borderline result defensible if anyone audits the source data.

Scope note

STARD applies to the CD-versus-UC discrimination analysis (Section 3.5). The remainder of the manuscript — correlation with activity indices, severity and behavior subgroups, and the one-year clinical course — falls outside the scope of a diagnostic accuracy study and is reported according to STROBE. It is reasonable to submit **both** checklists and to state in the cover letter that STARD covers the diagnostic accuracy component while STROBE covers the observational cohort component.

References

Bossuyt, P.M.; Reitsma, J.B.; Bruns, D.E.; Gatsonis, C.A.; Glasziou, P.P.; Irwig, L.; Lijmer, J.G.; Moher, D.; Rennie, D.; de Vet, H.C.W.; et al. STARD 2015: an updated list of essential items for reporting diagnostic accuracy studies. *BMJ* **2015**, *351*, h5527.

Full explanation and elaboration: https://www.equator-network.org/reporting-guidelines/stard/
